# The Influence of Prior Learning Experience on Pollinator Choice: An Experiment Using Bumblebees on Two Wild Floral Types of *Antirrhinum majus*

**DOI:** 10.1371/journal.pone.0130225

**Published:** 2015-08-11

**Authors:** Coline C. Jaworski, Christophe Andalo, Christine Raynaud, Valérie Simon, Christophe Thébaud, Jérôme Chave

**Affiliations:** 1 CNRS—Université Paul Sabatier, UMR 5174, Laboratoire Evolution et Diversité Biologique, Toulouse, France; 2 Université de Toulouse, INP-ENSIACET, LCA (Laboratoire de Chimie Agro-industrielle), Toulouse, France; 3 INRA, UMR 1010-CAI, Toulouse, France; Central China Normal University, CHINA

## Abstract

Understanding how pollinator behavior may influence pollen transmission across floral types is a major challenge, as pollinator decision depends on a complex range of environmental cues and prior experience. Here we report an experiment using the plant *Antirrhinum majus* and the bumblebee *Bombus terrestris* to investigate how prior learning experience may affect pollinator preferences between floral types when these are presented together. We trained naive bumblebees to forage freely on flowering individuals of either *A*. *majus pseudomajus* (magenta flowers) or *A*. *majus striatum* (yellow flowers) in a flight cage. We then used a Y-maze device to expose trained bumblebees to a dual choice between the floral types. We tested the influence of training on their choice, depending on the type of plant signals available (visual signals, olfactory signals, or both). Bumblebees had no innate preference for either subspecies. Bumblebees trained on the yellow-flowered subspecies later preferred the yellow type, even when only visual or only olfactory signals were available, and their preference was not reinforced when both signal types were available. In contrast, bumblebees trained on the magenta-flowered subspecies showed no further preference between floral types and took slightly more time to make their choice. Since pollinator constancy has been observed in wild populations of *A*. *majus* with mixed floral types, we suggest that such constancy likely relies on short-term memory rather than acquired preference through long-term memory induced by prior learning.

## Introduction

A large proportion of plants are insect-pollinated, and pollinator behavior may impact plant evolutionary dynamics. Since pollinators mediate pollen flow, they may strongly affect overall plant gene flow [1]. An abundant literature has focused on pollination syndromes, i.e. the reciprocal adaptation of the phenotype of both specialist partners [2]. Yet most flowering plants are visited by generalist pollinators, which may have a greater and more complex role in the eco-evolutionary dynamics of plant-insect interactions than specialist pollinators [3]. In the present study, we aim to document how floral signals influence pollinator preference depending on their individual experience, in bumblebees foraging on *Antirrhinum majus*.

Pollinator-induced selection relies on the capacity of the pollinator to discriminate floral types on the basis of floral signals, which are often multi-modal and complex [4]. Plant-pollinator interaction offers an excellent example of a signaling pathway that has evolved to cope with environmental complexity [5; 6]. Both visual signals, which include floral display, plant and flower morphology, flower color, and also chemical signals, such as floral scent, are especially critical in plant detection [4; 7]. Pollinators may select preferentially phenotypes that display particular traits. Most empirical demonstrations of such selection rely on correlations between floral trait variation, pollinator visit frequency, and plant fitness [8–11]. Also, differential flower choice across pollinator individuals or species contributes to maintaining phenotypic variability [12]. For instance, nocturnal moths mostly rely on floral scent to find the plant [13; 14], whereas hummingbirds mostly use visuals signals [13]. Complex density- and frequency-dependent patterns may occur, if pollinator preference for a particular phenotype is influenced by plant density [15]. In extreme cases, divergent selection may lead to speciation, with most different phenotypes having a higher fitness [16].

Many pollinators show constancy while foraging, i.e. they visit preferentially some floral types, ignoring valuable resources from other floral types, and thus induce preferential pollen transfer among similar individuals [17; 18]. This constancy in foraging behavior results from a learning process, which combines short-term and long-term memory [5]. Pollinators are able to acquire a preference, or an aversion for some floral signals by learning to associate these signals to a reward [19–21]. A rapidly expanding literature has used bumblebees and honeybees as biological model species to explore the cognitive processes involved during associative learning of a reward with simple visual and olfactory cues [22; 23]. Pollinators modify their behavior depending on their acquired experience, but also depending on environmental information, such as the variability of floral signals in the target species, the local diversity of floral species, their spatial arrangement, and background environment [5; 24; 25]. Semi-controlled experimental studies that integrate a certain degree of variability may help to predict foraging behavior of pollinators in natural conditions.

The *Antirrhinum majus* plant complex provides an excellent opportunity to study the role of generalist pollinators in reproductive isolation. Two inter-fertile subspecies differ in flower color, *Antirrhinum majus pseudomajus* with magenta flowers, and *Antirrhinum majus striatum* with yellow flowers. They are distributed in parapatry across the eastern half of the Pyrenees mountains, and a hybrid zone has been carefully studied [26]. Because both subspecies are exclusively insect-pollinated, pollinator-limited, and share pollinators [27], pollinators are likely to influence gene flow between subspecies, by inducing directional pollen transfer among plant individuals. First, bumblebees, which are the main pollinators of *A*. *majus* in the wild [28], appear to discriminate colors of both subspecies and of hybrids [29; 30]. Also, they are likely to use floral scent as a signal in this species [28]. Second, pollinator constancy during foraging has been observed in wild parental populations flanking the contact zone with one dominant floral type and few hybrids (C. Andalo personal observation). Also, plant female fitness measures suggest an assortative mating, which may result from directional pollen transfer through pollinator constancy [30]. Finally, a previous experiment has shown that color diversity of artificial inflorescences influences constancy in bumblebees foraging bouts; bumblebees preferentially visited and were more constant to the most common floral type, and their constancy was reinforced when the number of differently colored floral types increased [31].

In the present study, we aim at testing experimentally how the learning process of bumblebees affects their preference between *A*. *majus* subspecies, and how the differential use of plant signals (color vs. odor) affects their choice. We address the following questions: (i) how do the two subspecies differ in floral signals?; (ii) do bumblebees have an innate preference for one of the two floral type?; (iii) after training on one floral type, do they acquire a preference? and (iv) do preference patterns, if any, depend on plant signal availability?

## Materials and Methods

### Biological material

*Antirrhinum majus* (Plantaginaceae) is a hermaphroditic, self-incompatible, short-lived perennial, which produces annual inflorescences with personate, zygomorphic flowers. The two subspecies *A*. *m*. *pseudomajus* and *A*. *m*. *striatum* are related as sister clades [32; 33], and they differ by flower color. They occur parapatrically in the eastern part of the Pyrenees where they come into contact in a zone stretching 150 km along the Sierra del Cadi in Catalonia, Spain [26]. In *Antirrhinum*, the corolla is made of two lobes that close the flower, and only large bees are able to get access to the nectar (mostly *Bombus spp* and *Xylocopa violacea*, [28; 30]. Thus, insect pollination is obligatory in this system.

Plants used in this study were grown in a greenhouse. In October 2011, up to 50 mature individuals were sampled for seeds at each locality, in nine wild populations in the Pyrenees (Table 1). These localities are non protected, municipal areas and thus no specific permission was required to sample plant material. We collected only seeds of *A*. *majus*, which is not an endangered or protected species, and there are no other known protected or endangered species in the sampled localities. Seeds were grown into plants in the spring-summer 2012. Within populations, individuals were then hand-crossed at random; all individuals were crossed at least once. Next-generation seeds were exempt of maternal effects as all plants had grown in a common garden. In spring 2013, we used these next-generation seeds to grow plants that were used in our experiment with bumblebees. Growing conditions in the greenhouse were natural light with shading when solar radiation exceeded 800 W/m2, and temperature variations followed outdoor conditions but were constrained from 15 to 28°C. Plants were grown in individual pots (height 8 cm; diameter 10 cm) filled with universal compost, with no nutrient addition. Plants were automatically supplied with water twice a day and vertically grown with wood sticks. They were protected from possible pollinating and herbivorous insect species by a fine mesh material during their development and when they were not used for bumblebee training or behavioral tests.

**Table 1:** Description of the parental plant populations.

Acronym	latitude, longitude	Location	Altitude (m)	Subspecies	Sample size (floral scent)	Sample size (reflectance)	Plants used in learning sessions	Number of tests using plants of each locality	Description
BAN	42.488745, 3.124752	Banyuls-sur-Mer	25	*pseudomajus*	0	2	yes	0	Roadside bank (rocky)
BES	42.200420, 2.699175	Besalú	155	*pseudomajus*	3	6	yes	114	Stone walls in village
LUC	42.966881, 2.259513	Luc-sur-Aude	225	*striatum*	0	0	yes	18	Roadside bank and river-side bank (rocky)
LYS	42.831762, 2.209184	'Pierre-Lys' gorge	375	*striatum*	2	0	yes	30	Roadside bank (rocky / herbaceous)
MIJ	42.725279, 2.038218	Mijanès	1347	*striatum*	0	0	yes	0	Roadside bank (herbaceous
PAR	42.315363, 2.219561	Pardines	1098	*pseudomajus*	5	3	yes	74	Roadside bank (herbaceous)
PRA	42.404787, 2.479175	Prats-de-Mollo	761	*pseudomajus*	4	3	yes	126	Stone walls in village and rocky bank
THU	42.638797, 2.735808	Thuir	113	*striatum*	5	4	yes	25	Roadside bank (herbaceous)
VIL	42.591819, 2.365969	Villefranche-de-Conflent	531	*striatum*	3	1	yes	141	Bank (rocky and shrubs)

For our behavioral tests, we used *Bombus terrestris* (ssp. *terrestris*) bumblebees from six commercial colonies (Biobest, Orange, France). Colonies were fed with a nutritive sugar-based solution and with a mixture of pollen commonly used for beekeeping. They had had no contact with real flowers or other colored or scented rewarding source prior to the experiment, and were thus considered as flower-naive. Only worker bees were used in the experiment.

All experiments were performed at the CNRS Station of Experimental Ecology in Moulis, France, from July, 6^th^ to August 7^th^ 2014. Manipulations were conducted outdoors, except on rainy days where they were performed inside the greenhouse in a different compartment from the growing plants. Inasmuch as possible, we limited visual heterogeneities in the surroundings, which appeared either uniformly green (grass) to bumblebees or gray for indoor conditions. Throughout the experiment, climatic outdoor conditions were measured (greenhouse sensors Aria: temperature, rainfall, solar radiations). Due to the experimental time periods (from 5pm to nightfall; see Bumblebee behavioral tests section), we measured a large range of environmental conditions (temperature: 14–35°C; solar radiation: 0–700 W/m2), but we did not detect any effect of such variations (see Statistical analyses of bumblebee choicesee section).

### Characterization of plant signals of *A*. *majus* subspecies

Both visual and olfactory plant signals were measured on the same plants as those used in the behavioral tests on bumblebees (see Bumblebee behavioral tests section), except in cases when flowers had faded in between. This represented 77% of plants for floral scent (20 of 26 plants), and 31% of plants for color (8 of 26 plants). We also measured floral scent for three additional intact plants, and color for 11 additional plants that had been used to train bumblebees, assuming that nectar collection by pollinators does not significantly modify flower color. Floral scent was measured the same day as plants were used in bumblebee behavioral tests whenever possible, and plants were randomly sampled across subspecies each day. Flower color was sampled a few days after the tests.

#### Measure of flower color

Flower reflectance spectra were measured from 300 to 800 nm with a USB2000 spectrometer with a DH-2000 deuterium–halogen lamp and analyzed with the OOIBase32 software (Ocean Optics, Inc., Dunedin, FL). Measurements were carried out with the probe placed perpendicularly to a tangent to the surface, and reflectance data were expressed comparatively to a white standard disk (type WS; Labsphere, Congleton, United Kingdom). One to two flowers per plant were measured, on three 10 × 10 mm zones placed on a plane surface below the probe. The zones were chosen at three positions of the flower corolla for each flower: corolla tube, inferior and superior petals. Each measure was repeated twice. For each measure, color loci were calculated in a bee hexagon color space [31; 34–36], using the spectral sensitivity curves of the three receptors of the species *B*. *terrestris*, and the spectral reflectance of a natural green background positioned at the center of the hexagon. The hexagon representation illustrates how bees discriminate objects: the points more distant from the central position are better distinguished from the background color. The corolla tube, the less bright part of the flower corolla, was not different between *A*. *majus pseudomajus* plants and *A*. *majus striatum* plants, and thus not used to calculate the color loci of the plant. The color loci were defined as the average positions of the lower and upper corolla on all the flowers of the same plant.

#### Measure of floral scent

**Fig 1:**
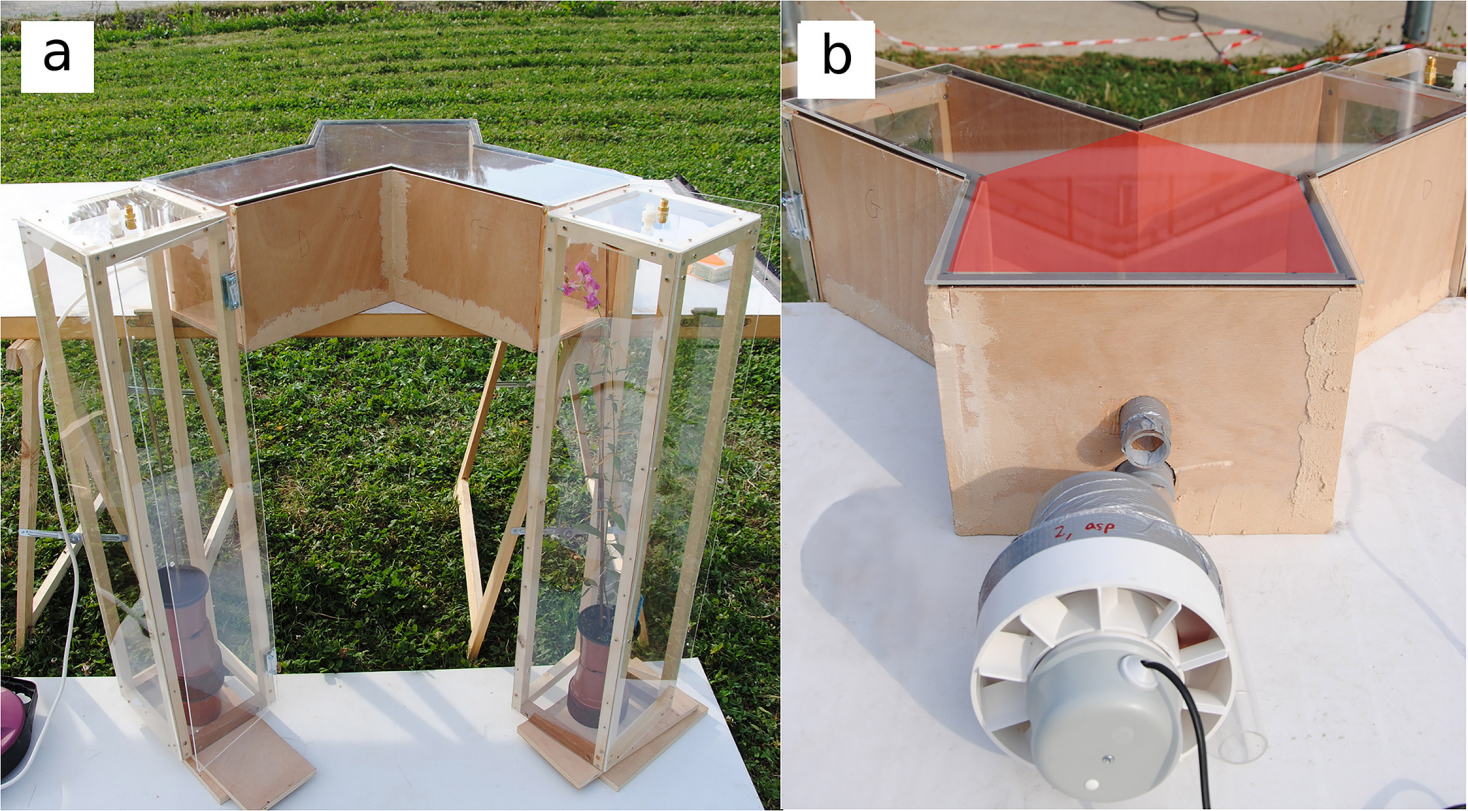
Y-maze apparatus used for bumblebee behavioral tests. (a) Position of plexiglas plant chambers facing the arm ends. The chamber at the left contains no plant (control: empty pot with a wooden stick). The chamber at the left contains a *A*. *majus pseudomajus* plant with four open flowers. Connectors for plant scent collection are visible on the top of the chambers. (b) Fan causing an air flow from arm ends to the main channel. The hole above the fan was used to put the bees inside the Y-maze. The Y-maze neutral zone is red-shadowed.

Floral scent was sampled on plants placed in chambers in the same position as during bumblebee behavioral tests (Fig 1A), between 12am and 3pm, which corresponds to the peak of emission intensity [28; 37]. Each chamber was connected to a 200 mL/min aspiring pump with Teflon-PTFE tubing. Volatile organic compounds (VOCs) were adsorbed on a 100 mg Tenax TA cartridge (60–80) placed between the chamber and the pump, during 15 min. This protocol optimizes the signal-to-threshold ratio without exceeding the breakthrough volume of each compound in the conditions of our experiment [38]. To discriminate VOCs emitted by the plant from possible environmental contamination, ambient air was sampled simultaneously to plants. Blank samples from the empty chambers and from the Y-mazes used for bumblebee tests were also collected. Sample cartridges were sealed with Teflon-coated brass caps immediately after collection, stored at 4°C, and analyzed at the most eight days later. Prior to sampling, all cartridges were cleaned by thermodesorption at 320°C, and analyzed by GC-FID (see next paragraph) to ensure the absence of contaminants on chromatograms. Throughout the sampling period, temperature (37 ± 8°C; range 27–52°C) and humidity (44 ± 13%; range 24–61%) of the floral headspace were monitored with a datalogger (EL-USB2-LCD+, Radiospare) placed inside the chamber.

VOC samples were thermodesorbed (cool trap -30 to 250°C at 40°C/s) with a Turbomatrix TD desorber (Perkin-Elmer, USA), and were analyzed with a gas chromatograph coupled with a mass spectrometer and a flame-ionization detector (GC-FID/MS, Clarus 500, Perkin-Elmer, USA) equipped with a DB-5 ms non-polar capillary column (5% phenyl-methylpolysiloxane; 30 m × 0.25 mm ID × 0.25 μm film thickness). Samples were heated a 35°C for 5 min, then to 160°C at 5°C/min and finally up to 220°C at 15°C/min and at 220°C for 10 min. The carrier gas was helium. Mass spectra were recorded in the electron impact mode at an ionization voltage of 70 eV and scanned from m/z = 35 to 450. VOCs were identified with their retention index relative to C5-C15 n-alkanes [39; 40]. The retention time of the n-alkanes were measured by injecting a mixture of C5-C12 standards, and using the retention time of C13-C15 n-alkanes measured in our plant samples, which are easy to identify by their mass spectra. The identity of the VOCs in the plant samples was confirmed by the mass spectra, which were matched at each peak with those from the NIST library (2005). Peaks that could not be identified were defined as morpho-molecules characterized by their retention index (Table B in S1 File); most were classified as fatty acid derivatives, and a few of them were classified as benzenoid compounds.

VOC abundance was quantified by its FID peak area, reported to external calibration of five pure standards (Sigma-Aldrich, France) that yielded the estimation of response factors *k* used in the calculation of VOC emission rates *E*. The protocol used for external calibration is detailed in S1 File. For each VOC, *E* (ng.min^-1^.flower^-1^) was obtained from the difference between the quantity of VOC measured in the floral scent and in ambient air: *E = (A*_*sample*_ *- A*_*air*_ *) / (k*.*t*.*N*_*f*_ *)*, with *A*_*sample*_ and *A*_*air*_ (area units) the peak areas of the floral scent sample and the control ambient air sample respectively, *k* ((area units).ng^-1^) the response coefficient, *t* (min) the sampling time, and *N*_*f*_ the number of flowers at anthesis of the plant. This equation is a simplified version of that used by [28], as we had no air purge of the chamber in our sampling method. Thus, the incident air flow was equal to the air flow carried through the collector cartridge. This is known that the emission rate of some VOCs can be altered by environmental conditions [41; 42], and especially photosynthetically active radiation and temperature, which led to the development of algorithms on the model VOC isoprene to standardize the emission rate in a range of environmental conditions [43]. However, the different VOCs in the floral blend of *A*. *majus* have very variable properties in terms of volatility and chemical class, thus we found that is was not relevant to use such a standardization. We did not find a strong impact of temperature or solar radiation on VOC emission.

Some peaks corresponded to molecules known to be atmospheric pollutants or non biogenic VOCs (ethylbenzene, styrene, naphtalene and phtalate derivatives, silicate and chlorate compounds), and other were quantified in large amounts in blank samples. These peaks were considered as contaminants, and they were excluded from the analysis. Rare peaks (present in less than 10% of the sampled plants) were also removed. We assumed that peaks could be detected if the absolute area exceeded 1000 area units, and the signal-to-noise ratio exceeded 10. Quantification thresholds were 10 ng for fatty acid derivatives, 0.75 ng for terpenic compounds, and 2.5 ng for benzenoid compounds, as estimated from the quantification thresholds of external standards (Table A in S1 File). Thus, VOCs below these thresholds were excluded from quantitative analyses (see next paragraph). Finally, peaks for which uncertainty on area estimation was greater than 10% in average were also excluded from quantitative analyses.

#### Statistical analyses of floral scent profiles

We measured 152 VOCs, after excluding four rare ones. We could identify 50 VOCs, and quantify 31 of them plus six unidentified VOCs. Differences in presence / absence of VOCs between subspecies were analyzed for the 152 VOCs separately, by comparing proportions of individuals of each species for which the VOC was recorded with the function prop.test (library stats; [44]). For the 37 quantified VOCs, we performed three analyses on the absolute quantities (in ng.min^-1^.flower^-1^), after a log-transformation and centering to reduce effects of variance heterogeneity due to difference in emission rates among VOCs, and to assign a relatively equivalent weight to each VOC. We first performed a principal component analysis of scent profiles to detect the most variable VOCs across plants. We then performed a partial least square-discriminant analysis (PLS-DA, R library mixOmics) to discriminate the scent profiles of *A*. *majus* subspecies. This analysis accounts for data sets with multi-collinear explanatory variables and a small number of samples compared to the number of explanatory variables; it also allows to test group separation [45; 46]. In PLS-DA, variables may be selected that explain most of the variance among samples; we selected explanatory VOCs for which the variable importance index was above one, a commonly used threshold. Third, we tested whether the variation of absolute emission rate in each VOC could be explained by subspecies with a two-sample *t*-test. Finally, we analyzed the emission rate ratios of the 37 quantified VOCs, when the absolute emission rate is normalized by the total emission rate of the 37 VOCs. We performed a correspondence discriminant analysis on the ratios.

All statistical tests in our study were performed in the R software (R Development Core Team 2014).

### Innate versus acquired preference of bumblebees for *A*. *majus* subspecies

#### Bumblebee training procedure

To test how the learning experience of the bumblebees may influence their preference between *A*. *majus* subspecies floral types, we generated three sets of individuals. The first two sets were trained to forage on plants of one subspecies only, and we then tested their choice between plants of different subspecies. The third set was composed of naive bumblebees and used as a control.

**Fig 2:**
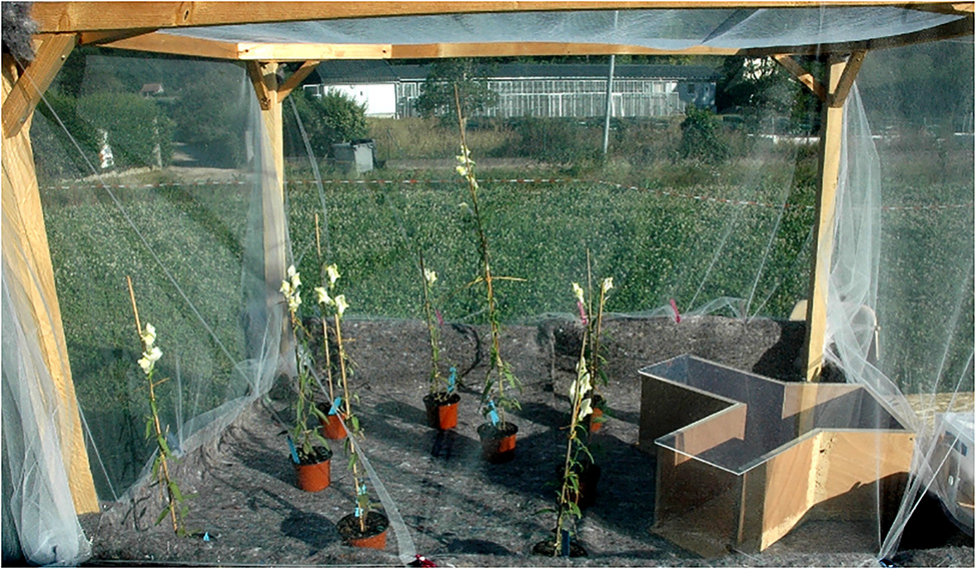
Flight cage used for bumblebee training procedure. Plants are of the subspecies *A*. *majus striatum* (yellow-flowered).

The learning assay was conducted as follows. Each day, two groups of five bumblebees were trained to foraging in a flight cage, one from 9am to 10am and the other from 10am to 11am. The most active bumblebees were isolated from their colony and introduced into the flight cage, through the Y-maze used in the behavioral tests (see below), but with arm ends open, to habituate them to the Y-maze environment. One colony was used each day, and we alternated the colonies. The flight cage consisted of a wooden frame of 1.6 × 1.2 × 0.9 m covered with a fine mesh (Fig 2). Six to eight plants that had from one to five flowers at anthesis each were randomly selected from the pool of available individuals from different geographical origins. They were randomly placed inside the cage, and were randomly permuted after half an hour. The total number of available open flowers was maintained as constant as possible throughout the training, but varied from 11 to 31 (mean 20 ± 1 SEM) due to flowering plant availability. Two groups of plants were used for the two sessions each day, and for three successive days, except the ones that had completed flowering, which were replaced. Flower nectar is naturally refilled in less than 24 h within an *Antirrhinum* flower (C. Suchet and E. Tastard, pers. obs.), thus all bumblebees had access to similar quantities of nectar throughout the experiment. During the training, bumblebees foraged freely. All foraging events were recorded, and classified as a visit when the bee landed on a flower without entering, or a nectar / pollen collection when the bee entered the flower. At the end of the training session, trained bees were placed together in an empty clean box until the late afternoon. They were supplied with water to avoid dehydration, but they were not provided with food to maintain optimal foraging conditions. We also trained the naive bees, using the same protocol as for bees trained on real flowers, but using artificial rewarding spots exempt of visual and olfactory cues.

#### Bumblebee behavioral tests

To test the joint effect of learning experience and plant signal availability on bumblebee choice, we used a factorial design, where we varied bumblebee experience (three modalities: *pseudomajus*-trained / *striatum*-trained / naive bees) and the type of plant signals (three modalities: color only, odor only, color and odor). All experiments were conducted in two Y-mazes simultaneously. The bees trained to foraging in a flight cage in the morning were exposed to a choice test in the late afternoon. We conducted choice tests between plants of different subspecies, and control tests between plants of the same subspecies. Control tests were conducted to reveal possible biases influencing bee behavior and bee ability to discriminate against two plants of the same subspecies. The two plants used in a test were selected to have the same number and arrangement of flowers along the stem, and had never been in contact with any insect before. To control for orientation, all tests were repeated with the same bees by varying the left / right position of the two plants at arm ends.

Y-mazes were constructed in wood covered by UV-transmitting Plexiglas (Fig 1). The main channel was 200 mm long, 283 mm wide and 200 mm high. The symmetric arms were 300 mm long, 200 mm wide and 200 mm high, and formed a 90° angle. This size is compatible with bee capacity to discriminate flowers, as an inflorescence of two-three flowers represents a spot of about 2 × 8 cm [47]. An air-flow system carried floral scent from arm ends to the main channel at 180mL/min, by air aspiration from a hole made in the front wall of the main channel (Fig 1B).

We controlled how floral scent was carried through the Y-maze for the three types of plant signals, by sampling air in each arm and at the entrance of the main channel with the same method used for floral scent sampling. For each VOC, the quantity measured in air samples inside the Y-maze was about 70% of the quantity measured in floral samples collected directly in the plant chamber. We did not detect a bias in scent emission: when the same plant was placed at each arm end, the quantities detected in each arm did not differ, thus floral scent plumes were considered equivalent between arms. Also, we did not detect a difference in scent quantities measured between the two Y-mazes. Finally, we verified that the olfactory plus visual treatment did not alter the VOC quantities compared with the olfactory signal only treatment. Quantities measured in the visual signal only treatment matched VOC quantities measured in ambient air around the Y-maze.

Bees were placed inside the Y-maze just above the air flow funnel (Fig 1B), through a UV-transparent plexiglas tube connected to a 100 × 100 mm landing area through a 35 mm-diameter hole. Arm ends were closed with three different sheets corresponding to the three types of plant signals. For the color treatment, sheets were Dura-Lar oriented polyester film. For the odor treatment, sheets were cardboard panels covered with fine mesh outside and occulted light or every other visual cue at the arm ends. For the treatment with both color and odor, sheets were Dura-Lar oriented polyester film, with a centered 30 mm-diameter hole located 20 mm below the roof, and covered by a 50 × 50 mm fine mesh to prevent bees from escaping. Plants were placed symmetrically in 200 × 200 × 900 mm chambers next to the arm ends (Fig 1A). Plant chambers consisted of a wooden frame covered by UV-transparent plexiglas except on the side next to the Y-maze arm ends, which was open. The Y-maze was virtually divided into three zones: left arm, right arm and neutral zone (red-shadowed on Fig 1B). A special attention was paid to the orientation of the Y-mazes during tests: we made sure that no direct sun radiation arrived by the arm ends, and that both arms had a similar light intensity.

Tests were conducted between 5pm and 8pm. Each bee was tested up to four times with at least 30 minutes between two successive tests. The first two tests were performed on the same plant signals, with plants inverted across arm ends to control for potential orientation bias, while the last two tests were performed on a different type of plant signals. Each test lasted five minutes from the time a bee was released into the Y-maze. Each time the bee crossed the border between zones of the Y-maze (right arm / left arm / neutral zone), the type of event, its duration and the type of movement were recorded. Tested bees mostly flew from one zone to the other, they more rarely walked. We also recorded when the bee was immobile. At the end of the five minutes, the tested bee was kept isolated in an aerated plexiglas tube before the next test. Between tests, Y-mazes were washed with 70°-ethanol and aerated for five minutes, then purged for another five minutes to stabilize the air flow. Between tests on different types of plant signals, Y-maze were washed with ethanol and aerated for 20 minutes.

#### Statistical analyses of bumblebee choice in behavioral tests

We analyzed bee preference in the Y-maze through their first choice. We expected learning experience to shift the bee innate preference between floral types toward the learned floral type [20]. Thus, we expected that bees trained on *pseudomajus* plants would tend to choose the *pseudomajus* plant, and that bees trained on *striatum* plants would tend to choose the *striatum* plant.

We found that naive bees tended to choose more often the left arm when two plants of the same subspecies were proposed (control tests). Since we do not know the origin of this bias, we controlled the impact of plant position in the statistical tests. This bias did not depend on the parental population of plants used in the test, nor on the Y-maze device.

We analyzed preference, i.e. the proportion of bees choosing first the *striatum* plant in choice tests depending on their learning experience and the type of plant signals. This convention (choice for the *striatum* plant) was arbitrary, and it allowed to test the three groups of bees simultaneously (naive bees, *pseudomajus*-trained bees, *striatum*-trained bees), and thus to assess how learning experience affects innate preference. We performed generalized-linear mixed models with the HLfit function of the R library spaMM [48], with a binomial error structure. Hive and bee nested within hive were specified as random factors. We first analyzed the whole dataset to detect possible differences in preference amongst the three groups of bees. Bee experience, the type of plant signals (color only, odor only, color and odor), and plant position (left: *striatum* plant and right: *pseudomajus* plant, vs. left: *pseudomajus* plant and right: *striatum* plant) were used as fixed effects. Starting with the most complex model with the interaction between the three factors, the most likely model was obtained by sequential removal of non-significant interactions of highest order. Significance of effects was assessed by a model comparison based on a log-likelihood ratio test.

To better disentangle the impact of plant position and type of plant signals, we analyzed the proportion of bees choosing first the *striatum* plant in the three data subsets of each group of bees separately. Fixed effects were the type of plant signals and plant position, and random effects were the same as previously. We tested sequentially the significance of interactions and effects. A significant preference for one floral type was assessed by the estimation of the intercept value (95%-confidence interval) on the best model for each of the three groups of bees independently. A 95%-confidence interval not containing 0 was interpreted as a significant preference or aversion.

Next, we tested the impact of other explanatory variables on first choice, such as test day, temperature during the test, number of flowers per plant used in the test, mean number of flowers visited during learning session (a proxy of learning strength), quality of plants proposed during the learning session (in terms of number of days of use). None of these variables had an impact on bee behavior in Y-maze (data not shown). To analyze bee constancy, we also used the ratio of time spent in each arm, and the ratio of moves of bees toward each arm. Similar results as for first choice were obtained and these results are omitted here.

Finally, we analyzed how bee training type and type of plant signals influenced the time to first choice. We used a Cox model using the 'coxme' R library. This model relies on a survival function modeling the probability that a particular event happens before a given time. In the survival function, we used the time before making a first choice and a two-level factor indicating the presence / absence of a first choice as the time and status variables. The fixed effects were bee experience and the type of available plant signals, and we used the same random factors as above. The most likely Cox model was obtained by removal of the non-significant interaction, and significance of fixed effects was assessed through a model comparison based on an analysis of variance.

## Results

### Characterization of signals of plant subspecies

**Fig 3:**
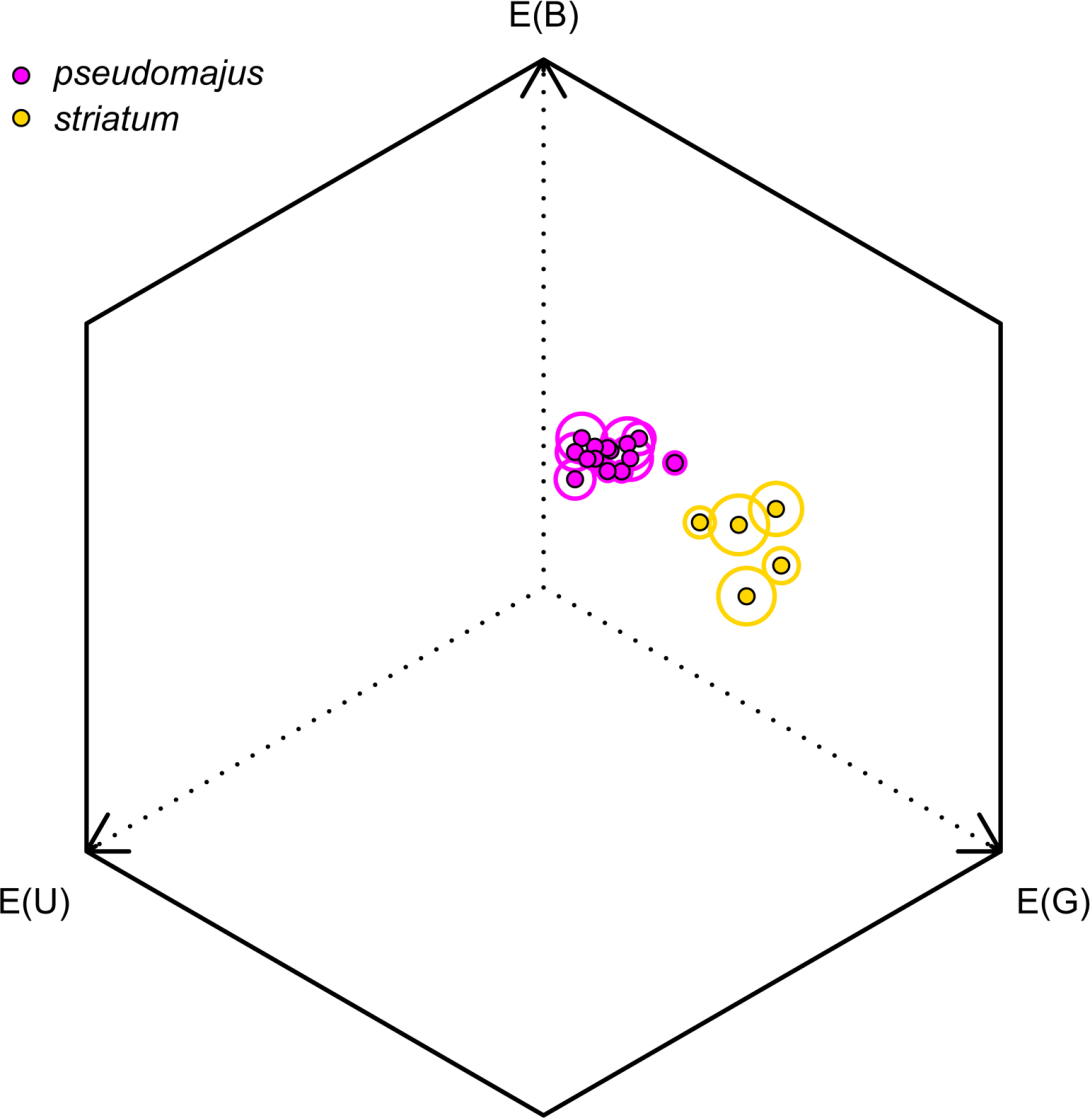
Color loci of plants used in bumblebee behavioral tests, in the *B*. *terrestris* color perception space. Mean position ± SE (circle) per plant, on measures taken on the upper and lower petal on one to two flowers per plant. Magenta dots: *pseudomajus* plants; golden dots: *striatum* plants.

Flower color was clearly different between *A*. *majus* subspecies in the bumblebee color perception space (Fig 3). In most of the plants, flower color was inhomogeneous, as shown by the standard error-circles represented on Fig 3. *A*. *m*. *striatum* plants were more distant from the center of the hexagon than *A*. *m*. *pseudomajus* plants, suggesting that *A*. *m*. *striatum* plants are better distinguished from a neutral, green background than *A*. *m*. *pseudomajus* plants.

**Fig 4:**
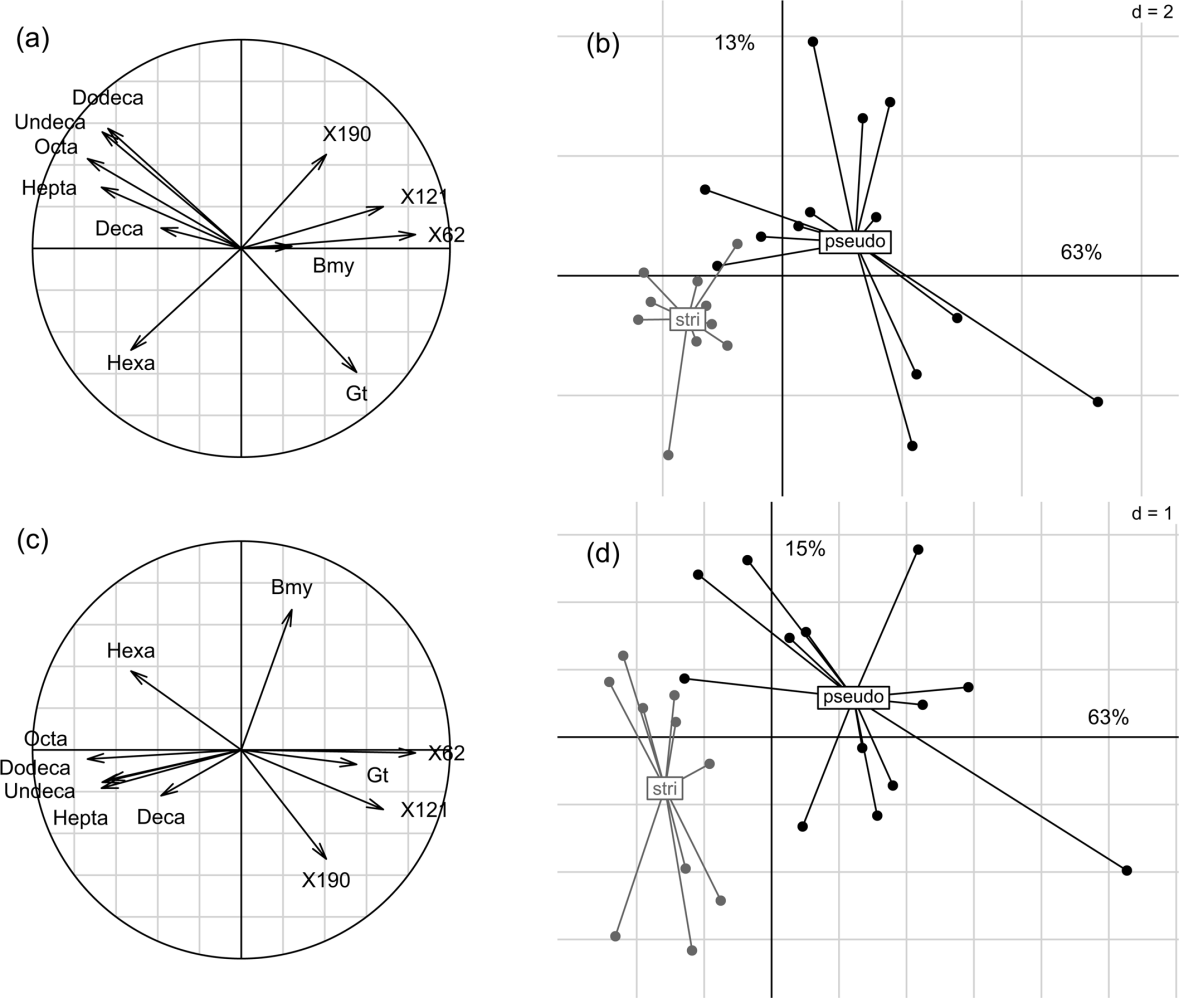
Partial least square-discriminant analysis (PLS-DA) on the 37 quantifiable VOCs in floral scent of *A*. *majus* subspecies (black: *A*. *m*. *pseudomajus*; gray: *A*. *m*. *striatum*). (a) correlation circle of the most discriminant VOCs, and (b) samples projection, on principal components 1 and 2. (c) correlation circle of the most discriminant VOCs, and (d) samples projection, on principal components 1 and 3. The variation explained by each of the principal components is shown as percentages along axes. Hepta': heptanal; 'Bmy': beta-myrcene; 'Hexa': hexanal; 'Octa': octanal; 'Undeca': undecanal: 'Dodeca': dodecanal; 'Deca': decanal; 'Gt': gamma-terpinene; #62, #121 and #190: unknown compounds.

Of the 152 VOCs measured in floral scent samples, three were found to occur significantly more in *A*. *m*. *striatum* than in *A*. *m*. *pseudomajus*: hexanal (Chi2 = 4.32, df = 1, *P* = 0.038); heptanal (Chi2 = 4.08, df = 1, *P* = 0.043), and one unidentified fatty acid derivative (Chi2 = 4.08, df = 1, *P* = 0.043). The PCA on absolute emission rates showed that *A*. *m*. *pseudomajus* had more variable emission rates than *A*. *m*. *striatum*, but the scent profiles did not differ across subspecies. Conversely, the PLS-DA based on VOC absolute emission rates successfully discriminated the floral scent profiles of plants of the two subspecies, with 11 VOCs explaining much of the variance among plants (Fig 4). Principal PLS-DA components in this analysis explained 63, 13 and 15% of the total variance, and the remaining components explained much lower levels of variance among samples. *A*. *m*. *striatum* was characterized by higher rates of aldehydes (hexanal, heptanal, octanal, decanal, undecanal and dodecanal) while *A*. *m*. *pseudomajus* was characterized by higher rates of terpenic compounds (beta-myrcene and gamma-terpinene) and of three unknown compounds. However, the predictive power of this discriminant analysis was low, with an error rate on group assignation exceeding 50%, likely due to high variability across floral scent profiles within both subspecies. Thus the two studied *A*. *majus* subspecies do not have significantly different floral scents.

These results were confirmed by a *t*-test run on the absolute emission rate of each of the 37 VOCs between subspecies (Table B in S1 File). Hexanal and heptanal were emitted at significantly higher rates, and octanal, undecanal and dodecanal at slightly higher rates in *A*. *m*. *striatum* than in *A*. *m*. *pseudomajus*. The other VOCs did not differ across subspecies. *A*. *m*. *striatum* plants tended to have higher total emission rates than *A*. *m*. *pseudomajus* plants, but this was not significant (Table B in S1 File).

Finally, similar patterns could be found in relative emission rates. Plants of *A*. *m*. *striatum* had higher relative amounts of aldehydes (heptanal, octanal, undecanal and dodecanal) and of methyl-undecene (isomer #1) and one unknown compound (#166). Plants of *A*. *m*. *pseudomajus* had higher relative amounts of beta-myrcene, acetophenone, and one unknown compound (#121). Together, all these differences in VOC emission rates between subspecies did not allow to unambiguously identify each plant as belonging to one subspecies.

### Innate versus acquired preference of bumblebees for *A*. *majus* subspecies

**Fig 5:**
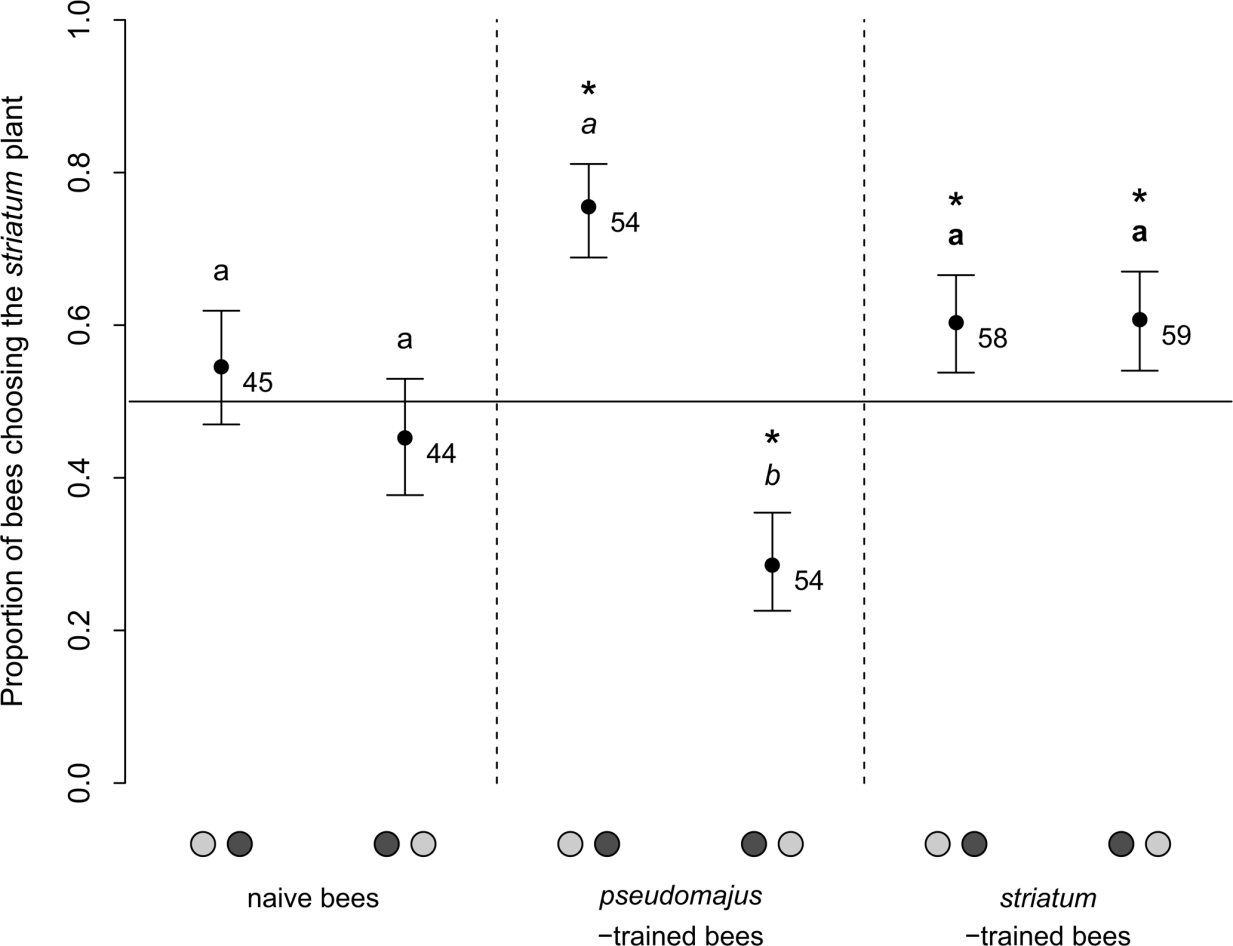
Proportion of bees choosing the *striatum* plant in the three groups of bees, and for the two plant positions in the Y-maze. Error bars are standard errors on model parameters estimated from non linear mixed model. Plant positions are specified on the x-axis by a left / right couple of spots (light gray: plant of the subspecies *striatum*; dark gray: plant of the subspecies *pseudomajus*). Letters show significance in log-likelihood ratio tests between proportions for each position, in the three groups of trained bees separately (naive bees: normal font; *pseudomajus*-trained bees: italic font: *striatum*-trained bees: bold font). Stars emphasize a significant deviation from random choice (proportion 0.5). Samples sizes are provided for each group.

Bee first choice was significantly influenced by their training experience (Table 2; Fig 5). Naive bees had no preference between subspecies (intercept estimate: 0.00 ± 0.22; 95CI: [-0.42; 0.45]), irrespective of plant position and or type of plant signals. *Striatum*-trained bees showed a significant though small preference for *striatum* plants (intercept estimate: 0.43 ± 0.20; 95CI: [0.01; 0.90]). This result held irrespective of plant position or the type of plant signals. This is consistent with the idea that bees use both visual and olfactory plant signals to discriminate and select their target plant. However, no reinforcement of preference for the learned floral type when both signals are available could be detected.

**Table 2:** Analysis of bumblebee preference between the two *A*. *majus* subspecies, on the basis of their first choice. Log-likelihood ratio tests were performed on generalized linear mixed model modeling the probability to choose the *striatum* plant, with bee experience, type of available plant signals, and position of the *striatum* plant as fixed effects, and hive and bee nested within hive as random factors. Each fixed effect was tested by controlling for the influence of all other fixed effects of equal or lower degree.

Explanatory factor	Log-likelihood ratio	Degrees of freedom	*P*-value
*1/ Entire dataset*			
Bee experience × Plant signals × Position	6.03	4	0.20
Bee experience × Position	14.4	2	0.00076 ***
Bee experience × Plant signals	0.750	4	0.95
Plant signals × Position	6.18	2	0.046 *
Bee experience	2.57	2	0.28
Position	9.82	1	0.0017 **
Plant signals	0.726	2	0.70
*2/ Naive bees*			
Plant signals × Position	4.21	2	0.12
Position	0.749	1	0.39
Plant signals	0.282	2	0.87
*3/ pseudomajus-trained bees*			
Plant signals × Position	7.81	2	0.020 *
Position	23.4	1	<< 0.001 ***
Plant signals	0.603	2	0.74
*4/ striatum-trained bees*			
Plant signals × Position	0.507	2	0.78
Position	0.00108	1	0.97
Plant signals	0.558	2	0.76

*** *P* < 0.001

** *P* < 0.01

* *P* < 0.05.

*Pseudomajus*-trained bees had no preference for either subspecies (intercept estimate: 0.082 ± 0.20; 95CI: [-0.38; 0.49]). We discovered that these bees chose the left arm of the Y-maze more often than random, and that this orientation bias was stronger when only olfactory signals were available, and weaker when only visual signals were available. However, we found no residual preference between subspecies for any of the three types of plant signals after removal of bias effect. Thus, our data do not support the shift of innate preference toward the learned floral type for *pseudomajus*-trained bees.

**Fig 6:**
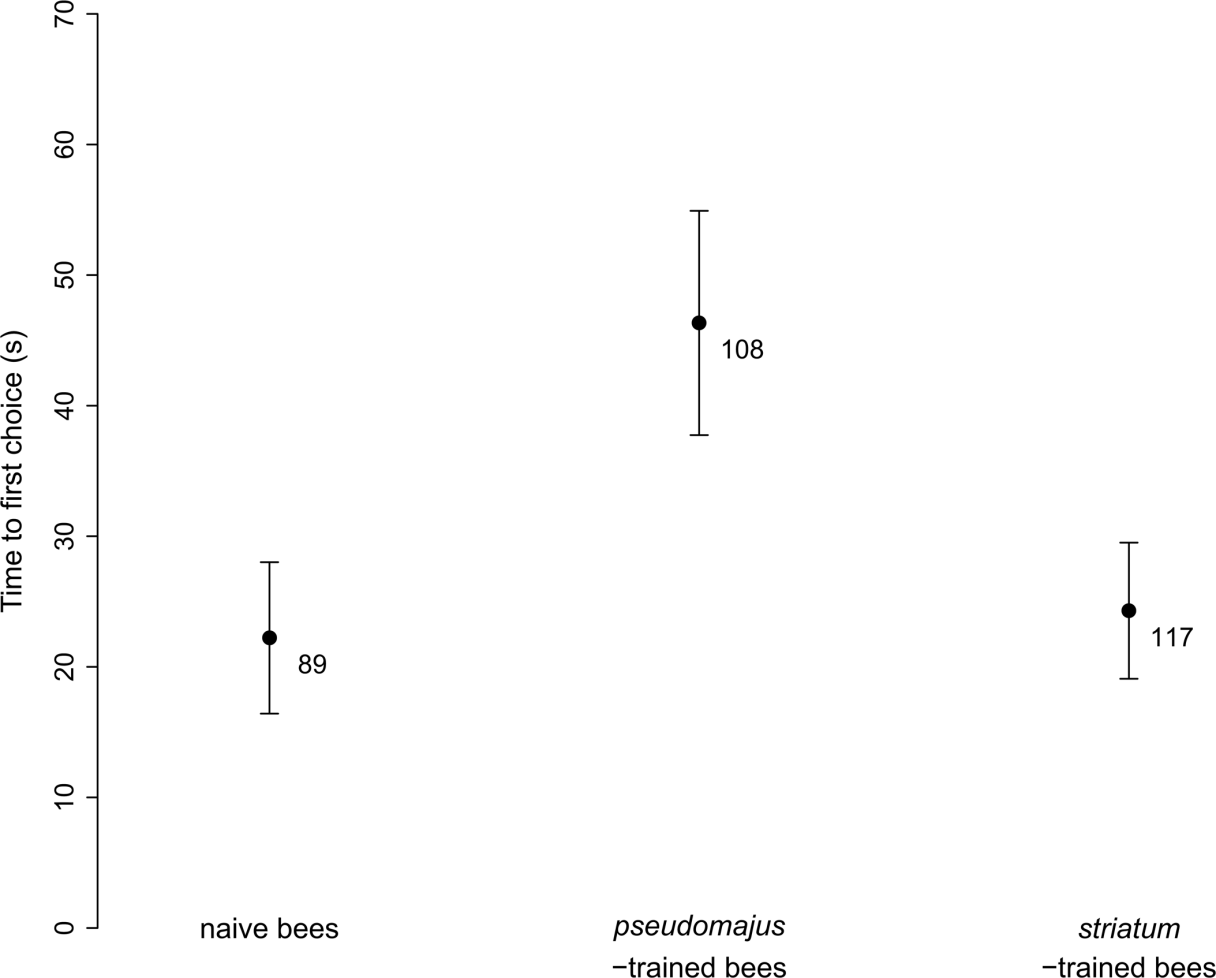
Time to first choice in the Y-maze experiment. Mean ± SE, for naive bees, bees trained on *pseudomajus* plants, and bees trained on *striatum* plants. Samples sizes are indicated below error bars.

We also analyzed possible factors influencing bee decision. We found that neither bee training experience nor the type of plant signals influenced the time to first choice (Table 3), although *pseudomajus*-trained bees tended to be slower at making a first choice between floral types than both naive bees and *striatum*-trained bees (Fig 6).

**Table 3:** Analysis of time to first choice between *pseudomajus* and *striatum* plants. Anova-tests were carried out on Cox models modeling the probability to make a choice after a certain time, with bee experience and type of available plant signals as fixed effects, and hive and bee nested within hive as random factors. Each fixed effect was tested by controlling for the influence of all other fixed effects of equal or lower degree.

Explanatory factor	Chi2	Degrees of freedom	*P*-value
Bee experience × Plant signals	6.98	4	0.14
Bee experience	2.09	2	0.35
Plant signals	0.670	2	0.72

## Discussion

We show that bumblebee preference for floral types of *A*. *majus* subspecies was influenced by their learning experience. Naive bumblebees did not have innate preference between the yellow-flowered subspecies *A*. *m*. *striatum* and the magenta-flowered subspecies *A*. *m*. *pseudomajus*. Bumblebees previously trained on *striatum* plants showed a preference for this subspecies in choice tests, but those trained on *pseudomajus* plants did not display a similar preference toward *pseudomajus* plants. Thus, acquired preference of bumblebees is influenced by the floral type used for learning. Finally, we did not observe a reinforcement of preference for the learned floral type when both visual and olfactory floral signals were available.

Our first goal was to carefully quantify the phenotype of the individual plants used in the experimental approach. Indeed, it is often assumed, rather than tested, that visible differences for humans imply distinguishable phenotypes for bees. We measured a clear difference in color between *A*. *majus* subspecies floral types (Fig 3), and this difference was distinguishable by bumblebees [29; 36]. However, we found that floral scent was not significantly different across subspecies (Fig 4). We found significantly higher emission rates of aldehyde compounds in *A*. *m*. *striatum* plants than in *A*. *m*. *pseudomajus* plants, but similar emission rates of benzenoid compounds, and high intra-group variability in the emission rates was measured for most VOCs. Aldehydes are known to be emitted mostly by plant leaves [49], thus differences in floral blends of the subspecies may be due to differences in leaf metabolism rather than to the floral blend. We measured leaf fresh weight on a reduced number of plants of both subspecies and did not find differences between subspecies, thus higher emission rates of aldehydes in *A*. *m*. *striatum* are unlikely due to higher leaf biomass. This result on floral scent is inconsistent with that of [28; 50], who measured a strong difference between subspecies, both in greenhouse and wild conditions, due to the emission of high quantities of benzenoid compounds in *A*. *m*. *pseudomajus* but not in *A*. *m*. *striatum*. Although we carefully explored this problem, we were unable to reproduce these results [28; 50]. One interpretation is that the biosynthesis pathway of benzenoids is under environmental control, and the difference of expression in wild plants of *A*. *majus striatum* compared with plants of *A*. *majus pseudomajus* is not maintained when the populations are self-crossed to remove maternal effects. It would be interesting to unveil differences in the regulation of the biosynthesis pathway of benzenoid compounds between *A*. *majus* subspecies, and whether these steps may indeed be triggered or down-regulated by environmental factors.

During our tests using the Y-maze, we contrasted the behavior of naive bumblebees and trained bees. We found that naive bumblebees did not have a preference for one of the floral types, irrespective of the type of plant signals that were available (Fig 5). This was unexpected since the literature suggests an innate preference for yellow flowers of closely related bee species [20; 51]. In our experiment, floral signals other than corolla color may alter the bumblebees innate preference for yellow flowers, possibly resulting in the observed absence of innate preference between *A*. *majus* subspecies.

The main goal of this study was to explore the processes by which pollinators acquire preference for certain floral types. Pollinator constancy is one possible mechanism contributing to the reproductive isolation between *A*. *majus* subspecies [26; 30; 31; 52], because such a foraging behavior reduces gene flow across different floral types. In a previous study, floral color diversity in a hybrid zone was simulated using artificial inflorescences [31]. This study showed a similar constancy for the dominant floral type, be it either yellow or magenta. In this light, the asymmetric effect of learning experience on preference between floral types in our study was unexpected. However, the learning process we imposed to bumblebees differs from what they experience when foraging freely among various floral types. We imposed a fixed time lag of several hours between training session and choice tests to bumblebees, so their acquired preference must have relied on long-term memory. Conversely, several studies showed constancy of different pollinator bee species within a foraging bout, which may mostly rely on short-term memory [31; 53; 54]. Short-term memory corresponds to instantaneous experience while foraging. It vanishes after a few visited flowers, and is more likely to be used than long-term memory in complex environments where the pollinator has to cope with multiple sources of information [5].

The asymmetric acquired preference across floral types suggests that the strength of associative learning was smaller for the *pseudomajus* floral type than for the *striatum* floral type (Fig 5). Preliminary data showed that *A*. *majus pseudomajus* flowers had higher or equivalent nectar quantities than *A*. *majus striatum* flowers in wild populations or in greenhouse-grown plants, with similar nectar composition and sugar ratio (C. Suchet, E. Tastard). Thus, differences in rewards between floral types is unlikely the cause of a difference in the strength of associative learning measured in our experiment.

Another explanation may be that *A*. *m*. *pseudomajus* floral signals are harder to perceive or less reliable than that of *A*. *m*. *striatum*. Yellow flowers are better discriminated than magenta flowers from background colors (Fig 3, and [55]), and bumblebees tend to prefer yellow flowers in wild populations with red and yellow floral types of several plant species [56; 57]. Thus flowers of *A*. *m*. *striatum* may be more easily associated to a reward in the long-term memory compared with that of *A*. *m*. *pseudomajus*. If floral signals are variable within floral types, they may trigger weak constancy as compared with constant floral signals [58]. The PCA on floral scent profiles suggests that floral scent is more variable in *A*. *m*. *pseudomajus* than in *A*. *m*. *striatum* plants. Conversely, our measures of corolla color show an opposite pattern. However, these measures do not account for contrast within the corolla, and neither for patterns of coloration, which both may guide bees to the resource and thus be involved in the associative learning [59; 60].

Finally, we measured an orientation bias on *pseudomajus*-trained bumblebees, but not on *striatum*-trained bumblebees. While this result is unlikely to strongly affect the pattern of acquired preference, as we controlled its impact with similar sample sizes for the two plant positions in the Y-maze, it could affect our ability to measure an acquired preference. This bias was not due to light or other differences in background visual cues between left and right arms, nor to the Y-maze used or other environmental variables measured. Thus, it still begs for an explanation, and it would be important to further explore the underlying mechanism. We further measured a slightly longer time before decision of *pseudomajus*-trained bumblebees than the two other groups (Fig 6). This suggests that they were more disorientated in the Y-maze.

On theoretical grounds, constancy is predicted to be stronger when floral types differ in multiple floral signals [61]. Although *A*. *majus* subspecies did not differ significantly in floral scent, *striatum*-trained bumblebees preferentially chose the *striatum* plant even when only olfactory signals were available. This suggests that bumblebees were able to discriminate floral types on the basis of both color and scent. Aldehydes were found at significantly higher rates in *A*. *m*. *striatum*, and they can be perceived by bumblebees [62; 63]. However, it would be surprising that such VOCs operate as attractants to pollinators, because they are often emitted by plants in reaction to herbivory, and are known to attract herbivores natural enemies [64]. They are also thought to reduce pollinator attractiveness, as they may signal herbivory-related damage [65]. Finally, bumblebees are highly sensitive to scent, and we may have failed to detect the VOC used as a signal by bumblebees to choose between floral types, due to limits in the floral scent sampling method.

Although our study does not directly relate to the impact of pollinator foraging behavior in natural populations, we here discuss possible implications. We showed that learning experience affects innate preference of bumblebees, but that the intensity of acquired preference depends on the learned floral type. In natural populations, this learning process could cause asymmetric constancy between *A*. *m*. *striatum* and *A*. *m*. *pseudomajus* floral types. Such an asymmetric foraging behavior could in turn cause a stronger assortative mating within yellow floral types than within magenta ones, when floral types are mixed, and reinforce the observed reproductive isolation between *A*. *majus* subspecies [26]. Complex context-dependent pollinator behaviors have been shown to play a role in floral isolation and hybridization in a number of cases (e.g. [15, 66–68]). In the *A*. *majus* wild populations, the bumblebee foraging behavior highlighted by our study would possibly result in *A*. *m*. *striatum* floral types to spread toward the *A*. *m*. *pseudomajus* parental populations at a contact zone, if such directional pollen flow is not strongly altered by the other minor pollinators of *A*. *majus*. A previous study on genetic introgression between *A*. *majus* subspecies at contact zones suggests reciprocal patterns of introgression rather than an unidirectional gene flow of *A*. *m*. *striatum* toward *A*. *m*. *pseudomajus* populations [69]. Especially, authors found that the largest hybrid zone has moved toward former *A*. *m*. *striatum* patches rather than *A*. *m*. *pseudomajus* patches. However, natural situations where both yellow and magenta floral types are mixed are likely more complex due to the presence of hybrid floral types. This may influence acquired preference and foraging behavior of pollinators [31], and result in complex patterns in plant maternal fitness [30]. Finally, long-term evolutionary trends of floral hybridization and isolation as measured through genetic data are likely to be influenced by variation in pollinator availability, which may be stochastic [70–72].

Bridging the gap between field observations and experimental approaches has been a longstanding challenge in ecology. Over past years the study of the mechanisms of pollinator cognitive processes has moved towards increasingly detailed analysis of brain functioning, and this has been elucidated in part based on lab experiments [22; 63]. However, the consequences of these processes for pollinator behavior in the wild remains elusive. In the present study, we attempted to bridge this gap in assessing the influence of floral signal variability, variability of environmental conditions and learning experience on pollinator decision. Expectedly, our results are not as striking as those obtained under fully controlled conditions, but they shed light on the generalization of other experimental results.

Our study demonstrates a complex interaction between floral phenotypes and acquired pollinator preference, when controlling the relative importance of visual versus olfactory floral signals. These floral signals are predicted to be some of the most important floral signals for pollinator attraction [4; 7]. Both types of signals shape acquired preference of pollinators, and the strength of acquired preference depends on the floral type.

## Supporting Information

S1 File Supplementary information on floral scent analysis. (DOC)

S2 File Color data. X,Y coordinates of color reflectance data of flowers of *A*.*majus* subspecies in the *B*. *terrestris* hexagon color space.
(XLS)

S3 File Scent data. Presence/absence data and absolute emission rates of VOCs measured in floral scent samples of *A*. *majus* plants.
(XLS)

S4 File Bee data. Data of bumblebee behavioral tests and training sessions.
(XLS)
