# Supplementary material for: The Influence of Prior Learning Experience on Pollinator Choice: An Experiment Using Bumblebees on Two Wild Floral Types of Antirrhinum majus
Source: PLoS One. 2015 Aug 11;10(8):e0130225. doi: 10.1371/journal.pone.0130225 (PMC4532467; doi:10.1371/journal.pone.0130225)
Supplement: S1 File — (DOC) [file pone.0130225.s001.doc]

**S1 File. Supplementary information on floral scent analysis**

**Protocol used for external calibration of floral scent emission rates.**

The calibration was carried out by injecting 50 µL of standards diluted in methanol (purity 98%) at known concentrations into Tenax cartridges, which were desorbed and analyzed in the same conditions as the floral scent samples (see main article). This yielded a response factor *k* for each standard, which is the correlation coefficient between the quantity of injected standards, and peak area (Table S1). We used the following response factors *k*: 2625 (area units).ng^-1^ as a mean between isovaleraldehyde and nonanal, which was used for all compounds assigned to the family of fatty acid derivatives; 5700 (area units).ng^-1^ as a mean between α-pinene and β-pinene, which was used for all terpenic compounds; and 4817 (area units).ng^-1^ for acetophenone, which was used for all benzenoid compounds.

**Table A**. Characteristics of chemical standards used for external calibration of floral scent emission rates.

| **VOC** | **Purity**  **(%)** | **Detection threshold**  **(ng)** | **Quantification threshold**  **(ng)** | **Calibration range**  **(ng)** | **Response factor *k***  **((area units).ng^-1^)** |
| --- | --- | --- | --- | --- | --- |
| isovaleraldehyde  nonanal  α-pinene  β-pinene  acetophenone | 97  95  98  99  > 99.5 | 1.0  1.0  0.01  0.01  0.25 | 10  10  0.75  0.75  2.5 | 10 – 1400  10 – 1400  0.75 – 8500  0.75 – 8500  2.5 – 28400 | 2925 ± 100  2326 ± 80  5845 ± 55  5560 ± 71  4817 ± 44 |

**Table B**. Statistics of *t*-tests and mean values of absolute emission rates for both *A. majus* subspecies. *t*-tests were done on log-transformed absolute emission rates of each VOC separately to account for significant differences in mean emission rates across *A. majus* subspecies. Unknown compounds are identified by their retention index RI.

| **VOC** | ***t*-value** | **Degrees of freedom** | ***P*-value** | **Mean emission rate**  **(± SE) in *A. m. pseudomajus***  **N = 13**  **(ng.min^-1^.flower^-1^)** | **Mean emission rate**  **(± SE) in**  ***A. m. striatum***  **N = 10**  **(ng.min^-1^.flower^-1^)** |
| --- | --- | --- | --- | --- | --- |
| Unknown #3 (RI = 688)  1-heptene  3-methylbutanal  tropilidene  hexanal  p-xylene  nonane  heptanal  alpha-pinene  camphene  benzaldehyde  unknown #62 (RI = 966)  hemimellitene  sabinene  beta-pinene  beta-myrcene  p-menthene  octanal  D-limonene  *cis*-beta-ocimene  *trans*-beta-ocimene  gamma-terpinene  acetophenone  nonanal  allo-ocimene  methyl-undecene (is. 1)  methyl-undecene (is. 2)  unknown #121 (RI = 1163)  dodecane  decanal  undecanal  unknown #166 (RI = 1368)  tetradecane  unknown #173 (RI = 1407)  dodecanal  *cis*-caryophyllene  unknown #190 (RI = 1479)  **Total** | -0.33  -1.59  -1.30  -0.06  -3.23  -1.47  -1.07  -3.31  -1.37  -1.65  -1.18  1.87  -1.38  -0.46  -1.28  0.84  0.05  -2.75  -0.76  -0.73  -1.13  0.18  0.88  -1.04  -1.39  -0.90  -0.08  1.23  -0.77  -2.04  -2.38  -0.84  -1.83  -1.34  -2.32  -0.95  0.37  -1.45 | 18.6  18.4  18.1  19.3  20.8  20.7  21.0  12.6  16.0  16.0  21.0  12.0  17.0  19.6  16.4  17.1  19.6  12.5  20.9  16.9  17.6  20.0  17.2  20.7  14.6  15.4  18.3  20.6  20.8  20.9  13.1  16.9  13.7  13.0  13.3  16.6  18.7  19.3 | 0.74  0.13  0.21  0.95  0.0040 **  0.16  0.30  0.0058 **  0.19  0.12  0.25  0.086  0.19  0.65  0.22  0.41  0.96  0.017 *  0.46  0.48  0.27  0.86  0.39  0.31  0.18  0.38  0.94  0.23  0.45  0.054  0.033 *  0.41  0.090  0.20  0.037 *  0.36  0.72  0.16 | 0.059 ± 0.0032  0.018 ± 0.018  0.014 ± 0.005  0.13 ± 0.09  0.12 ± 0.05  0.024 ± 0.008  0.14 ± 0.07  0.20 ± 0.07  1.29 ± 0.48  0.0079 ± 0.0067  0.25 ± 0.10  0.0099 ± 0.0073  0.0059 ± 0.0054  0.039 ± 0.017  0.25 ± 0.10  0.33 ± 0.10  0.12 ± 0.04  0.84 ± 0.29  0.24 ± 0.10  0.014 ± 0.009  0.21 ± 0.08  0.0088 ± 0.068  1.87 ± 0.60  1.24 ± 0.51  0.0026 ± 0.0019  0.0056 ± 0.0050  0.14 ± 0.07  0.10 ± 0.05  0.35 ± 0.19  0.96 ± 0.52  1.02 ± 0.31  0.017 ± 0.012  0.10 ± 0.04  0.0017 ± 0.0017  0.77 ± 0.25  0.020 ± 0.017  0.30 ± 0.10  **11.2 ± 2.9** | 0.0081 ± 0.0049  0.11 ± 0.04  0.10 ± 0.04  0.11 ± 0.05  0.76 ± 0.29  0.052 ± 0.025  0.063 ± 0.021  0.38 ± 0.07  1.75 ± 0.87  0.023 ± 0.015  0.34 ± 0.07  0  0.0080 ± 0.0034  0.047 ± 0.016  0.30 ± 0.15  0.18 ± 0.06  0.11 ± 0.05  1.24 ± 0.25  0.48 ± 0.24  0.039 ± 0.028  0.29 ± 0.20  0.0045 ± 0.0030  2.37 ± 1.16  1.77 ± 0.63  0.021 ± 0.012  0.022 ± 0.014  0.24 ± 0.10  0.024 ± 0.024  0.23 ± 0.08  1.11 ± 0.29  1.66 ± 0.34  0.12 ± 0.07  0.13 ± 0.03  0.011 ± 0.006  1.09 ± 0.26  0.054 ± 0.027  0.30 ± 0.18  **15.6 ± 4.3** |
